# Nitrous anhydrase activity of carbonic anhydrase II: cysteine is required for nitric oxide (NO) dependent phosphorylation of VASP in human platelets

**DOI:** 10.1080/14756366.2021.1874946

**Published:** 2021-01-28

**Authors:** Dimitrios Tsikas, Stepan Gambaryan

**Affiliations:** a Institute of Toxicology, Core Unit Proteomics, Hannover Medical School, Hannover, Germany;; b Sechenov Institute of Evolutionary Physiology and Biochemistry, Russian Academy of Sciences, Petersburg, Russia

**Keywords:** Carbonic anhydrase, mass spectrometry, nitrous anhydrase activity, H_2_^18^O

## Abstract

The carbonic anhydrase (CA) family does not only catalyse the reversible hydration of CO_2_ to bicarbonate, but it also possesses esterase and phosphatase activity. Recently, bovine CA II and human CA II have been reported to convert inorganic nitrite (O=N-O^−^) to nitric oxide (NO) and nitrous anhydride (N_2_O_3_). Given the ability of NO to mediate vasodilation and inhibit platelet aggregation, this CA II activity would represent a bioactivation of nitrite. There are contradictory reports in the literature and the physiological role of CA II nitrite bioactivation is still disputed. Here, we provide new experimental data in support of the nitrous anhydrase activity of CA II and the key role L-cysteine in the bioactivation of nitrite by CA II. Using washed human platelets and by measuring VASP phosphorylation we provide evidence that exogenous nitrite (10 µM) is bioactivated to NO in a manner strongly depending on L-cysteine (100 and 200 µM). The process is not inhibitable by acetazolamide, a potent CA inhibitor. The contradictory results of recently published studies in this area are thoroughly discussed.

## Introduction

The “inherent” catalytic activity of the carbonic anhydrase (CA, EC 4.2.1.1) family is the reversible hydration of CO_2_ to bicarbonate (HCO_3_^−^) (R1) which is inhibited by several classes of drugs, among which sulphonamides (RSO_2_NH_2_) such as acetazolamide. In addition, CA isoforms are known for long time to possess esterase and phosphatase activity and to exert these activities by the same mechanism. Recently, bovine and human CA II have been reported to convert inorganic nitrite (O=N-O^−^) to nitric oxide (NO)^1^ and to nitrous anhydride (N_2_O_3_)^2^^,^^3^. While authentic NO can be directly detected, there is no analytical method to detect native N_2_O_3_ in biological samples thus far. N_2_O_3_ may exist in four very labile forms: one form is ON-NO_2_ (ON^●^ + N^●^O_2_ ↔ ON-NO_2_) without the nitrous anhydride structure N-O-N; the other three forms are isomeric O=N-O-N=O^4^. The results of our previous work^2^^,^^3^ suggest that N_2_O_3_ has the structure O=N-O-N=O. This structure is used in the present work throughout.

The conversion of O=N-O^−^ to NO is a redox reaction and implies the provision of one electron by the CA II. Yet, such a mechanism is difficult to reconcile for redox-inactive Zn^2+^-containing CA II. A nitrous anhydrase activity of CA II seems more realistic, because such a reaction requires protons which can be easily provided by the enzyme (R2). Given the ability of NO to mediate vasodilation and inhibit platelet aggregation, and the potential of N_2_O_3_ to nitrosate different functionalities of biomolecules to form for instance *S*-nitrosothiols (RSNO; (R3)), *N*-nitrosoamines (RNHNO; (R4)), the nitrous anhydrase CA II activity would represent a bioactivation of nitrite.

(R1) $${\text{CO}}_{2}+H_{2}O↔{{\text{HCO}}_{3}}^{-}+H^{+}$$

(R2) $$O=N-O^{–}{+}^{–}O-N=O+2\;H^{+}↔O=N-\text{OH}+\text{HO}-N=O↔O=N-O-N=O+H_{2}O$$

(R3) $$O=N-O-N=O+\text{RSH}↔O=N-O^{–}+\text{RSNO}+H^{+}$$

(R4) $$O=N-O-N=O+{\text{RNH}}_{2}↔O=N-O^{–}+\text{RNHNO}+H^{+}$$

Nitrite and nitrate are metabolites of NO endogenously produced from L-arginine by the catalytic action of NO synthase (NOS). Exogenous nitrite and nitrate are also present in foods and drinking water and represent a considerable source of NO bioactivity via bacterial nitrate reductase activity in mouth and gut flora. Pharmacological nitrite, an NOS-independent source of NO, has protective effects in the cardiovascular system by decreasing blood pressure and inhibiting platelet activation^5^. Administration of nitrite has been reported to lower blood pressure and to inhibit platelet aggregation in vivo in humans and mice^6^^,^^7^. In contrast, nitrite added to washed human or mice platelets *in vitro* was found not to activate soluble guanylyl cyclase (sGC) and not to inhibit platelet activation^7^^,^^8^. We supposed that the different effects of nitrite in vivo and in washed platelets *in vitro* might be connected with the absence of extra-cellular L-cysteine in washed platelet preparations. In our experiments with washed human platelets we tested this hypothesis and used L-cysteine at physiological concentrations.

The NO/sGC/protein kinase G (PKG) system is one of the most powerful mediators of platelet inhibition. Activation of PKG results in phosphorylation of multiple substrates involved in platelet inhibitory mechanisms. Vasodilator-stimulated phosphoprotein (VASP) is one of the well-established PKG substrates and is often used as a marker of the activation of this kinase^9^. In our experiments with washed human platelets, PKG activation was monitored by measuring VASP phosphorylation at Ser239 (P-VASP^Ser239^) using phospho-specific antibodies. It is worth mentioning that human platelets contain CA I and CA II, but they do not contain NOS^10^.

There are contradictory reports in the literature regarding the above mentioned newest CA II activities and even a disputation of a physiological role of CA II in the bioactivation of nitrite^11–14^. Here, we provide new experimental data in support of the nitrous anhydrase activity of CA II.

## Materials and methods

### Experiments with isolated CA II

NaNO_2_ was obtained from Riedel-de-Haën (Seelze, Germany). 2,3,4,5,6-Pentafluorobenzyl bromide (PFB-Br), L-cysteine, bovine CA II and ^15^ N-labelled nitrite (Na^15^NO_2_; declared >98 atom % ^15 ^N) were from Sigma-Aldrich (Steinheim, Germany). H_2_^18^O (declared isotopic purity >98 atom % ^18 ^O) was purchased from Medical Isotopes (Pelham, New Hampshire, USA). The GC column Optima-17 (15 m × 0.25 mm i.d., 0.25-µm film thickness) and the conical glass vials were obtained from Macherey-Nagel (Düren, Germany). GC-MS analyses were performed on a ThermoElectron DSQ mass spectrometer coupled directly to a Thermo-Electron Focus gas chromatograph (GC) equipped with an autosampler AS 3000 (ThermoElectron, Dreieich, Germany). Quantification was performed by selected-ion monitoring (SIM) of ions with mass-to-charge (*m/z*) ratios of *m/z* 46 for ^16 ^O=N-^16^O^‒^, *m/z* 48 for ^16 ^O=N-^18^O^‒^ and ^18 ^O=N-^16^O^‒^, *m/z* 50 for ^18 ^O=N-^18^O^‒^, and *m/z* 47 for the internal standard (^16 ^O=^15^N-^16^O^−^, ^15^NO_2_^−^). The dwell time was 50 ms for each ion.

The previously reported GC-MS method for nitrite and nitrate had been originally validated for 100-µL sample aliquots^15^^,^^16^. This method was adapted to 10-µL aliquots and validated for the CA microassay which involves the use of H_2_^18^O needed to prepare the aqueous buffer^3^. For highest derivatization yield of nitrite, the incubation time was 5 min. Under these conditions, nitrate cannot be quantified accurately^15^. We compared in parallel the methods using 100-µL (in quadruplicate) and 10-µL aliquots (in duplicate) of nitrite solutions in 100 mM Tris-HCl buffer, pH 7.4, in H_2_^16^O at added nitrite concentrations of 0, 2.5, and 5.0 µM. These concentrations were chosen because they were expected to be formed in experiments in H_2_^18^O-Tris buffer. The relative standard deviation values were 2.9% and 2.4%, 0.5% and 1.0%, and 3.1% and 4.3%, respectively. The regression equations obtained from linear regression analyses of measured nitrite (*y*) versus added nitrite (*x*) were *y* = 0.752 + 0.645*x*, *r*^2^=0.9945 for the 100-µL samples and *y* = 0.800 + 0.668*x*, *r*^2^=0.9998 for the 10-µL. The *y*-axis intercepts reveal nitrite concentrations in the Tris-HCl buffer of 0.75 µM and 0.80 µM, respectively. The slope values of the regression equations of 0.645 and 0.668 are very close indicating almost complete agreement (96.5%) between the procedures in the investigated concentration range.

### Experiments with washed human platelets

Human washed platelets were prepared as reported previously^17^ with small modifications. Blood was obtained from healthy volunteers, after they gave informed consent, according to our institutional guidelines and the Declaration of Helsinki. Studies using human platelets were approved by the Sechenov Institute of Evolutionary Physiology and Biochemistry of the Russian Academy of Sciences (IEPHB RAS) (Study No.3–03; 02.03.2019). Blood was collected into ACD solution (12 mM citric acid, 15 mM sodium citrate, 25 mM D-glucose, all final concentrations). Platelet rich plasma (PRP) was obtained by 5 min centrifugation at 330 × g; then collected PRP was centrifuged for 10 min at 430 × g, the pelleted platelets were washed in CGS buffer (120 mM sodium chloride, 12.9 mM trisodium citrate, 10 mM D-glucose, pH 6.5), and suspended in modified HEPES buffer (140 mM sodium chloride, 20 mM sodium bicarbonate, 5 mM potassium chloride, 1 mM magnesium chloride, 5 mM D-glucose, 10 mM HEPES, pH 7.4) to a final cell concentration of 3 × 10^8^ platelets/mL. After 15 min rest in a 37 °C water bath, washed platelets (100 µL/tube) were used in the experiments. Platelets were incubated with 10 µM sodium nitrite, at 100 µM or 200 µM concentrations of L-cysteine. The NO donor, sodium nitroprusside (SNP, 1 µM) was used as a positive control. Acetazolamide (200 µM) was used for inhibition of CA activity, sGC activity was inhibited by 20 µM ODQ (both inhibitors were pre-incubated for 10 min). All chemicals in these experiments were obtained from Sigma-Aldrich (Steinheim, Germany).

For Western blot analysis, after incubation with the mentioned compounds, 100 µL of 3x Laemmli buffer were added to each tube, proteins were separated by SDS-PAGE and transferred to nitrocellulose membranes. The membranes were incubated with Phospho-VASP^Ser239^ Nanotools (Teningen, Germany) or actin (Cell Signalling, Frankfurt/am Main, Germany) primary antibodies overnight at 4 °C. For visualisation of the signal, goat anti-mouse (for Phospho-VASPSer239) or anti-rabbit (for actin) IgG conjugated with horseradish peroxidase were used as secondary antibodies followed by electrochemiluminescence detection (GE Healthcare). Blots were analysed densitometrically using NIH Image J software for uncalibrated optical density.

### Experiments with purified S-[^15^N]nitrosoalbumin

In order to investigate the effects of protein-bound Cu^2+^ and free L-cysteine on the formation of nitrite from a high-molecular-mass *S*-nitrosothiol we performed investigations with freshly prepared and purified *S*-[^15^N]nitrosoalbumin without bound Cu^2+^ (i.e. S^15^NALB) and with Cu^2+^ bound to S^15^NALB (i.e. Cu^2+^-S^15^NALB), as well as with native albumin without bound Cu^2+^ (i.e. ALB) and with Cu^2+^ bound to albumin (i.e. Cu^2+^-ALB) as described elsewhere^18–20^. For this, each 6-ml aliquots of freshly prepared plasma obtained by centrifugation (10 min, 800 × g, 4 °C) of EDTA-anticoagulated blood donated by a healthy young volunteer were used. These samples were used to prepare ALB and S^15^NALB. Aliquots (2.5 ml) of the plasma samples were incubated for 10 min at room temperature with 1 mM CuSO_4_ to prepare Cu^2+^-ALB and Cu^2+^-S^15^NALB. All preparations were extensively purified using first Sephadex PD10 columns. Then eluates were extracted using 5-ml HiTrapBlue Sepharose affinity columns and washed with 100 ml of 67 mM potassium phosphate buffer, pH 7.0 (buffer A) in order to eliminate remaining free Cu^2+^. Albumin species were eluted with 5 ml of 67 mM potassium phosphate buffer, pH 7.0, that contained 1.5 mM KCl (buffer B), the eluates were reduced to about 1-ml using a stream of nitrogen gas and used subsequently without delay.

Each 50-µL aliquots of S^15^NALB and Cu^2+^-S^15^NALB were incubated for 2 min at room temperature in buffer A in the absence (-Cys) or in the presence (+Cys) of L-cysteine at a final concentration of 100 µM. Each 50-µL aliquots of S^15^NALB were incubated with ALB or Cu^2+^-ALB followed by their incubation in the absence (-Cys) or in the presence (+Cys) of L-cysteine at a final concentration of 100 µM as well. In order to quantify ^15 ^N-nitrite in the samples, they were spiked with ^14 ^N-nitrite as the internal standard at a final concentration of 10 µM and derivatized as described previously^15^. Each four 100-µL aliquots of all samples were treated with 10 µL of PFB-Br and 400 µL of acetone. Subsequently, the samples were incubated for 5 min at room temperature to generate the PFB derivatives of ^15 ^N-nitrite and ^14 ^N-nitrite with the latter serving as the internal standard. PFB derivatives were extracted with toluene (300 µL) and 1-µL aliquots thereof were analysed by GC-MS in the SIM mode: *m/z* 47 for ^15 ^N-nitrite and *m/z* 46 for ^14 ^N-nitrite. The concentration of ^15 ^N-nitrite in the samples was calculated by multiplying the peak area ratio of *m/z* 47 to *m/z* 46 by 10 µM, which was the concentration of the internal standard in the samples.

## Results

**Figure 1.**
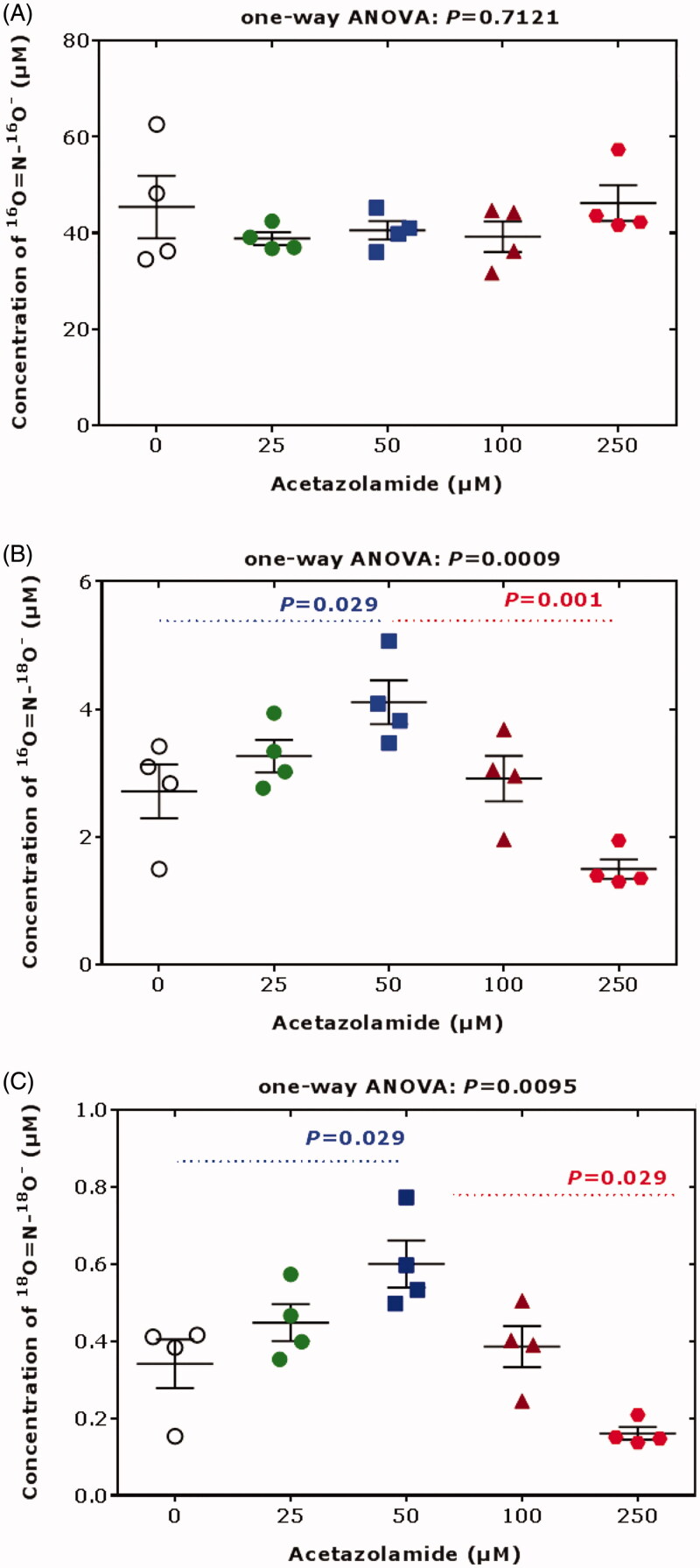
Concentrations of the nitrite species (A) ^16 ^O = N-^16^O^‒^, (B) ^16 ^O = N-^18^O^‒^ and ^18 ^O = N-^16^O^‒^, and (C) ^18 ^O = N-^18^O^‒^ upon incubation of sodium nitrite (100 µM) with bovine erythrocytic CA II (5 mg/mL, 172 µM) and acetazolamide (0, 25, 50, 100, 200 µM) for 10 min in 100 mM Tris buffer, pH 7.4, prepared in ^18 ^O-water (>98% ^18 ^O). The experiment was performed as described elsewhere^3^. Data are shown as mean with standard deviation from separate quadruplicate incubations. Statistical analysis was performed by one-way ANOVA and Mann-Whitney test.

We demonstrated that bovine CA II (bCA II) catalyses the incorporation of ^18 ^O into nitrite from H_2_^18^O used to prepare the pH-neutral buffered bCA II solution (Figure 1). Previously, we found that acetazolamide does not affect bCA II-catalyzed formation of NO from nitrite in the presence of exogenous L-cysteine at pH 7.4^3^. In that study we also found that acetazolamide itself did not affect the ^18 ^O-incorporation from H_2_^18^O into nitrite in the absence of bCA II,^3^ suggesting exclusive involvement of bCA II in this process. In the absence of L-cysteine, acetazolamide can modulate bCA II-catalyzed incorporation of ^18 ^O from H_2_^18^O into nitrite in a concentration-dependent manner, leading to a higher ^18 ^O-incorporation at 50 µM acetazolamide and to a lower ^18 ^O-incorporation at 250 µM acetazolamide compared to no use of acetazolamide (Figure 1). The acetazolamide concentration of 50 µM is pharmacologically relevant in human plasma (mean *C*_max_, 120 µM^21^). The acetazolamide concentration of 250 µM occurs in human urine upon ingestion of a single 250-mg acetazolamide tablet by healthy subjects^22^. The results of Figure 1 suggest that acetazolamide may modulate the nitrous anhydrase activity of bCA II in a non-linear concentration-dependent manner. A similar phenomenon was observed for the esterase activity of human CA II (hCA II) towards carbohydrate-based sulfamate inhibitors^23^.

**Figure 2.**
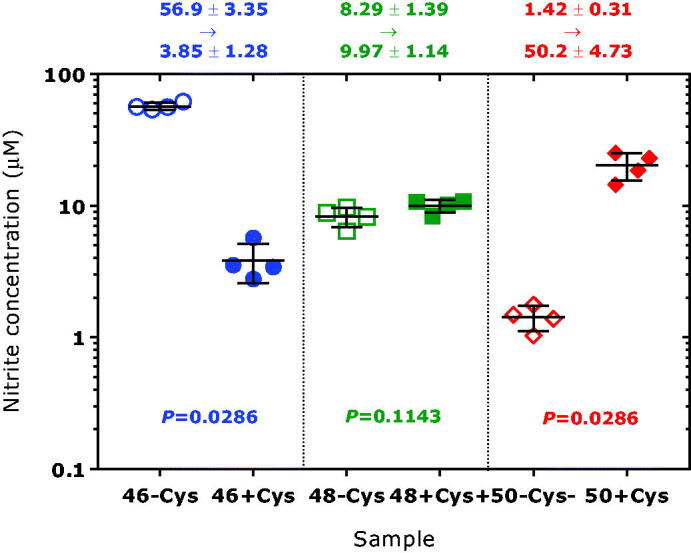
Concentrations of the nitrite species (left panel) ^16 ^O = N-^16^O^‒^, (middle panel) ^16 ^O = N-^18^O^‒^ and ^18 ^O = N-^16^O^‒^, and (right panel) ^18 ^O = N-^18^O^‒^ upon incubation of sodium nitrite (100 µM) with bovine erythrocytic CA II (5 mg/mL, 172 µM) in 100 mM Tris buffer, pH 7.4, prepared in ^18 ^O-water (>98% ^18 ^O) in the absence of L-cysteine (-Cys) or in the presence of L-cysteine (+Cys) at 100 µM. The experiment was performed as described elsewhere^3^. Data are shown as mean with standard deviation from four independent incubations. Statistical analysis was performed by one-way ANOVA and Mann-Whitney test.

By using this method we found that bovine and human erythrocytic CA II catalyses the incorporation of ^18 ^O atoms from H_2_^18^O used to prepare the 100 mM Tris-HCl buffer, pH 7.4, into nitrite, suggesting intermediate formation of nitrous anhydride and its subsequent hydrolysis to form singly and doubly ^18 ^O-labelled nitrite with *m/z* 48 and *m/z* 50, respectively^3^. The presence of exogenous L-cysteine at a concentration of 100 µM was found to potentiate the nitrous anhydrase activity (incorporation of ^18 ^O)^3^. In present study we also measured the concentration of remaining (unlabeled) nitrite, i.e. ^16^O_2_-nitrite (*m/z* 46). The results of these investigations are illustrated in Figure 2. To our surprise, we observed a strong decrease in the concentration of ^16^O_2_-nitrite (*m/z* 46), i.e. from 56.9 ± 3.35 µM without L-cysteine to 3.85 ± 1.28 µM in the presence of 100 µM L-cysteine (*P* = 0.029). bCA II-mediated consumption of nitrite was observed in the absence of external L-cysteine, albeit to a smaller extent (e.g. 70.3 ± 4.8 µM vs. 54.2 ± 11.4 µM; by 23%). In the absence of bCA II no such decrease or formation of *m/z* 48 and *m/z* 50 in the 100 mM Tris-HCl buffer, pH 7.4, was observed. These findings have not been reported previously and suggest a considerable consumption of nitrite in a manner dependent on bCA II and L-cysteine. Possible reactions could involve nitration of certain CA II amino acid residues including Cys^205^, the single L-cysteine residue of CA II, and various L-tyrosine residues (8 Tyr residues in CA II^24^). As *tris*(hydroxymethyl)aminomethane was used at 100 mM to prepare the Tris buffer, we cannot exclude a reaction of nitrous anhydride with *tris*(hydroxymethyl)aminomethane (p*K*_a_, 7.81) to form *tris*(hydroxymethyl)-*N*-nitroso-aminomethane which would simulate nitrite consumption. In the absence of L-cysteine the molarity of Tris buffer (10 − 100 mM) had not effect of nitrite consumption^3^. In this context it is notable that *tris*(hydroxymethyl)aminomethane can also react with *p*-nitrophenylacetate^25^ which is commonly used to measure the esterase activity of CA.

**Figure 3.**
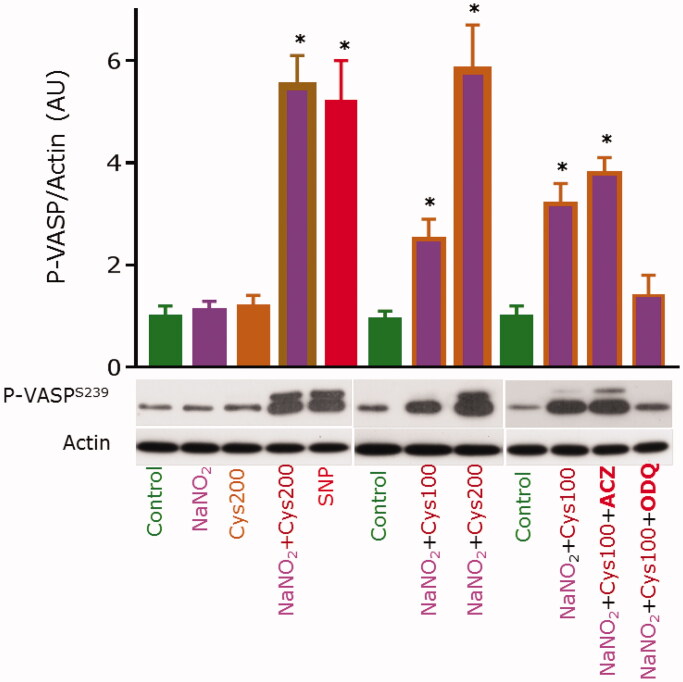
Cysteine is required for the bioactivation of nitrite to NO in human platelets. Washed human platelets (3 × 10^8^/mL, 100 µL) were incubated with indicated compounds. The concentration of NaNO_2_ was 10 µM where added. L-Cysteine (Cys) was added at final concentrations of 100 µM or 200 µM. The incubation time was 5 min. Sodium nitroprusside (SNP) was used at 1 µM (1 min) and served as positive control. Some samples were pre-incubated for 10 min with the CA inhibitor acetazolamide (ACZ) at 200 µM, or with the sGC inhibitor ODQ at 20 µM; then NaNO_2_ and Cys were added and the samples were incubated for 10 min. All samples were processed for Western blot analysis of VASP^Ser239^ phosphorylation, with actin blots serving as loading control. Blots were scanned and analysed densitometrically using NIH Image J software for uncalibrated optical density (graph). Results are presented as mean with standard deviation from quadruplicate analyses. Asterisk (*) indicate *p* values < 0.05 compared to control. Statistical analysis was performed by one-way ANOVA and Student’s *t*-test.

Incubation of washed human platelets with 10 µM sodium nitrite or 100 µM L-cysteine alone did not result in an increase of the P-VASP/actin ratio as compared to control (Figure 3). The intra-platelet P-VASP/actin ratio increased almost 6-fold upon of co-incubation of platelets with 10 µM sodium nitrite and 200 µM L-cysteine (Figure 3). This increase was very similar to that observed by incubating the washed human platelets with 1 µM sodium nitroprusside (SNP, Na_2_[Fe(CN)_5_NO]). The P-VASP/actin ratio was dependent upon the added L-cysteine concentration. At 100 µM acetazolamide, the P-VASP/actin ratio observed from 10 µM sodium nitrite/100 µM L-cysteine was almost the same as in the absence of acetazolamide, apparently suggesting no involvement of platelet CA. In contrast, pre-incubation of washed human platelets with ODQ, an inhibitor of sGC^26^, inhibited the phosphorylation of VASP, suggesting conversion of sodium nitrite to NO.

**Figure 4.**
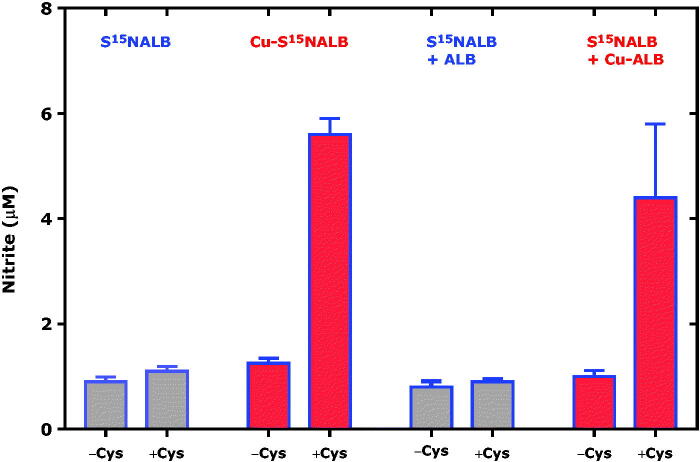
L-Cysteine, copper ions bound to albumin (Cu-ALB) and *S*-[^15^N]nitrosoalbumin (Cu-S^15^NALB) are required for the formation of ^15 ^N-nitrite from S^15^NALB. The initial concentrations were 13 µM for S^15^NALB and 15 µM for Cu-S^15^NALB. The concentration of free L-cysteine added to the samples (+Cys) was 100 µM. The concentration of free L-cysteine in the samples non-spiked with L-cysteine (-Cys) is unknown. The protein concentration in ALB, Cu-ALB, S^15^NALB and Cu-S^15^NALB was about 300 µM.

Human serum albumin (ALB) has a single cysteine moiety that does not form intra-molecular disulphide bridges. This cysteine moiety (Cys34) is accessible to modifications including *S*-nitrosation by nitrous anhydride and alkyl nitrites to form *S*-nitrosoalbumin (SNALB). SNALB itself is not an NO donor, but can release NO indirectly by means of free L-cysteine and intermediate formation of *S*-nitrosocysteine (CysSNO) via *S*-transnitros(yl)ation reactions.^18–20^ In the present study we investigated whether Cu^2+^ ions bound to albumin (Cu-ALB) and S^15^NALB (Cu-S^15^NALB) may modulate the L-cysteine-dependent release of NO from SNALB in buffer of neutral pH. Because nitrite (i.e. ^14 ^N-nitrite) is abundantly present as a contamination we used commercially available ^15 ^N-nitrite, i.e. the nitrite analog labelled with the stable isotope ^15 ^N, of which the natural abundance is 0.36%. The results of this experiment are shown in Figure 4.

Addition of L-cysteine at 100 µM to virtually Cu^2+^-free S^15^NALB alone or in the presence of Cu^2+^-free ALB in potassium phosphate buffer did not result in elevated formation of ^15 ^N-nitrite. In contrast, addition of L-cysteine at 100 µM to Cu^2+^-bound S^15^NALB (Cu-S^15^NALB) alone or in the presence of Cu^2+^-containing ALB (Cu-ALB) did result in elevated formation of ^15 ^N-nitrite by 37% and 34%, respectively. These results indicate that free L-cysteine interacts with Cu-S^15^NALB and S^15^NALB − Cu-ALB. However, the results of Figure 4 do not exclude the possibility that the incubation of S^15^NALB with Cu-ALB have resulted in formation of Cu-S^15^NALB prior to the addition of L-cysteine.

## Discussion

In the sections that follow below, we discuss the results of the present study and previous studies from our groups in the context of the results reported by other groups with respect to CA II functions as nitrous anhydrase or nitrite reductase. The discussion addresses mechanistic aspects and results from *in vitro* and in vivo studies.

### Mechanistic aspects: CA II as nitrous anhydrase and nitrite reductase

Pickerodt et al. 2019^13^ investigated the effects of inhaled sodium nitrite (iNaNO_2_) alone and in combination with intravenously administered acetazolamide (ivACZ), a strong inhibitor of hCA II (IC_50_, 12 nM^26^), in pigs under hyperoxic and hypoxic conditions. Based on missing differences of single iNaNO_2_ and combined administration of iNaNO_2_ and ivACZ with respect to various parameters, the authors concluded that CA is not a relevant nitrite reductase or nitrous anhydrase in the lung^13^. We agree with these authors that there is no convincing structure within the active site of the Zn^2+^-containing hCA II (i.e. Zn-hCA II), notably including the central redox-inactive Zn^2+^, or elsewhere in this protein capable of supporting one-electron reduction of nitrite (N oxidation state, +3) to form NO (N oxidation state, +2): ON^III^O^‒^ + e^‒^ + H_2_O → N^II^O + 2 OH^‒^. Hence, Zn-hCA II does not possess a nitrite reductase activity in contrast to the first proposal by Aamand et al. 2009^1^ and the later study by Nielsen & Fago^27^ based on the measurement of NO in their studies. Our groups found by a NO-selective electrode that Zn^2+^-containing bovine CA II (Zn-bCA II) and Zn-hCA II do not generate NO from nitrite in aqueous buffer of neutral pH in the absence or in the presence of acetazolamide or dorzolamide^2^^,^^3^. Aamand et al. 2009^1^ and Nielsen & Fago 2015^27^ reported that Zn-CA II-induced generation of NO from nitrite, which was even increased by the CA inhibitor dorzolamide stronger at pH 5.9 compared to pH 7.3. A possible explanation for these findings could be NO formation from the chemical reaction of nitrite with the amine (NH_2_) group of the sulphonamide functionality of dorzolamide at pH 5.9. Such reactions have been reported for many sulphonamides of which the amine group is quite acidic and easily nitrosable by nitrous acid (ONOH) and N_2_O_3_ to finally decompose to NO^28^. An alternative explanation could be that the Zn-CA II preparation used in that study contained contaminating Cu^2+^ (see below). On the other hand, we do not agree with Pickerodt et al. 2019^13^ that CA II does not exert nitrous anhydrase activity to produce N_2_O_3_ from the labile intermediate nitrous acid: ONO^‒^ + ONO^‒^ + 2 H^+^ → 2 [ONOH] → N_2_O_3_ + H_2_O. The conversion of ONO^‒^ to N_2_O_3_ is not a redox reaction and does not require any electrons, but solely protons that can be provided by CA II even in the absence of CO_2_/HCO_3_^‒^ (R1). The results by Aamand et al.^1^ are also supportive of a nitrous anhydrase activity of bCA II leading to the gaseous thus far undetectable N_2_O_3_ which may in part decompose to NO^4^, thus producing signals when measured by a NO-electrode or by chemiluminescence.

Andring et al.^11^ reported that Zn-CA II, a cytosolic CA isozyme, does not exhibit nitrate reductase or nitrous anhydrase activity. We agree that Zn-CA II is not a nitrate reductase, but we disagree that Zn-CA II is not a nitrous anhydrase. Our groups^2^^,^^3^ found by liquid chromatography-tandem mass spectrometry (LC-MS/MS) that bCA II and hCA II generated in the presence of glutathione (GSH) *S*-nitrosoglutathione (GSNO); we also found that in the presence of exogenous L-cysteine, bCA II and hCA II mediated NO formation as measured by a NO-sensitive electrode most likely via intermediate formation of *S*-nitrosocysteine (CysSNO), a potent NO donor. GSNO and CysSNO exert both cGMP-dependent (via NO release) and cGMP-independent (via *S*-transnitrosylation) biological activities, including vasodilation and inhibition of platelet aggregation.^29–31^ The results reported in the present study using washed human platelets, nitrite and L-cysteine are in support of intra-platelet Zn-CA II-mediated conversion of nitrite to NO via intermediate formation of CysSNO from N_2_O_3_ and L-cysteine, but not via reduction of nitrite to NO. We have demonstrated by mass spectrometry-based proteomic studies that CA II is physiologically present in platelets of healthy humans (35,000 copies/platelet).^10^

Our studies suggest that upon hCA II-induced conversion of nitrite to N_2_O_3_, this anhydride has the potential to develop NO-related bioactivity by subsequent redox-independent reactions. One mechanism may involve *S*-nitrosylation by N_2_O_3_ of the Cys^205^ moiety of Zn-hCA II to form hCA II-Cys^205^-S-N = O (R5a). Cys^205^ is in close proximity to the active site of Zn-hCA II. N_2_O_3_ could also *O*-nitrosylate the acidic aromatic hydroxyl (OH) group of the Tyr^7^ residue of Zn-hCA II to form Zn-hCA II-Tyr^7^-O-N = O (R6a). Tyr^7^ is in close proximity to His^64^ in the active site of Zn-hCA II as well. Although Tyr^7^ is not required for efficient hydratase and esterase activity, it is considered important for the stabilisation of the protein^32^^,^^33^. Subsequent reactions of Zn-hCA II-Cys^205^-S-N = O and Zn-hCA II-Tyr^7^-O-N = O with cytosolic GSH (R5b, R5c, R6b, R6c) would yield GSNO. In addition, N_2_O_3_ generated by the cytosolic Zn-hCA II could react with other cytosolic thiols including GSH and L-cysteine (R7, R8) and membranous thiols^34^. The reaction of GSNO with L-cysteine (CysSH) would form CysSNO (R9). CysSNO can be actively transported through cell membranes and donate NO^34^.

Nitrite reacts both with water and CO_2_, and these reactions can be modulated by Zn-hCA II^35^. The p*K*_a_ value of H_2_CO_3_ during its formation in aqueous solution is 3.5^36^, i.e. it is much lower than the generally assumed value of 6.4. Zn-hCA II-dependent N_2_O_3_ formation from nitrite and CO_2_ could occur via nitritocarbonate/nitritocarbonic acid (O=N-O-COO^-^/O=N-O-COOH) across the proton transfer shuttle (R10).

(R5a) $$\text{hCA}\;\text{II}-{\text{Cys}}^{205}-\text{SH}+O=N-O-N=O\to \text{hCA}\;\text{II}-\text{Cys}-S^{205}-N=O+O=N-O^{–}+H^{+}$$

(R5b) $$\text{hCA}\;\text{II}-{\text{Cys}}^{205}-S-N=O+G-S-H↔\text{hCA}\;\text{II}-{\text{Cys}}^{205}–\text{SH}+G-S-N=O$$

(R5c) $$\text{hCA}\;\text{II}-{\text{Cys}}^{205}-S-N=O+\text{Cys}-S-H↔\text{hCA}\;\text{II}-{\text{Cys}}^{205}–\text{SH}+\text{Cys}-S-N=O$$

(R6a) $$\text{hCA}\;\text{II}-{\text{Tyr}}^{7}-\text{OH}+O=N-O-N=O\to \text{hCA}\;\text{II}-{\text{Tyr}}^{7}-O-N=O+O=N-O^{–}+H^{+}$$

(R6b) $$\text{hCA}\;\text{II}-{\text{Tyr}}^{7}-O-N=O+G-S-H\to \text{hCA}\;\text{II}-{\text{Tyr}}^{7}-\text{OH}+G-S-N=O$$

(R6c) $$\text{hCA}\;\text{II}-{\text{Tyr}}^{7}-O-N=O+\text{Cys}-S-H\to \text{hCA}\;\text{II}-{\text{Tyr}}^{7}-\text{OH}+\text{Cys}-S-N=O$$

(R7) O=N−O−N=O+G−S−H→O=N−O–+G−S−N=O

(R8) O=N−O−N=O+Cys−S−H→O=N−O–+Cys−S−N=O

(R9) G−S−N=O+Cys−S−H↔G−S−H+Cys−S−N=O

(R10) $$O=N-O-\text{COOH}+O=N-O^{–}+\to O=N-O-N=O{+}^{–}\text{OCOOH}$$

### Potential nitrite-dependent physiological and pharmacological roles of carbonic anhydrase

Besides mechanistic aspects, other important issues should also be considered in the context of nitrite-dependent physiological and pharmacological roles of Zn-hCA and are discussed below.

In the study by Pickerodt et al.^13^, sodium nitrite (NaNO_2_, 450 mg, 6.5 mmol) was inhaled by juvenile male 24-kg weighing pigs. This corresponds to a dose of 18.8 mg/kg body weight and is about 16 times higher than the reported maximum tolerated dose of iNaNO_2_ in healthy humans^37^. After 1 h of hypoxia, arterial blood nitrite concentration increased 35-fold after iNaNO_2_ alone and even 48-fold after co-administration with ivACZ^13^. After 2 h of hypoxia, arterial blood nitrite concentration was only 8 µM in both cases, suggesting a short elimination half-life of nitrite. Mean arterial blood pressure decreased almost equally with iNaNO_2_ alone (79 to 60 mmHg) and in co-administration of acetazolamide (79 to 56 mmHg) after 1 h of hypoxia^13^. The average fractional excretion of NO (FENO) increased from 6 ppb at baseline to 46 ppb at 1 h, and fall to 27 ppb at 2 h, and to 20 ppb at 3 h upon iNaNO_2_ alone; the corresponding FENO values for iNaNO_2_+ivACZ were 6 ppb, 22 ppb, 13 ppb, and 11 ppb, i.e. constantly (almost by 50%) lower^13^. In that study, considerable differences between iNaNO_2_ and iNaNO_2_+ivACZ were also found for the respiratory rate. Despite considerable differences in some pharmacodynamic parameters, for instance 1) the lower FENO values (by 24, 14 and 9 ppb after 1 h, 2 h and 3 h of hypoxia) and 2) the mean pulmonary arterial pressure (MPAP) of the combination of iNaNO_2_+ivACZ compared to iNaNO_2_ alone (by 9 and 8 mmHg after 2 h and 3 h of hypoxia), the authors concluded that CA is not relevant to the bioactivation of nitrite^13^. We think that these observations may suggest that at the very high dose of iNaNO_2_ used in the study^13^, iNaNO_2_ may have attenuated/inhibited the nitrous anhydrase CA activity in the lungs. Acetazolamide has been demonstrated to increase the excretion of endogenous and exogenous nitrite and to a lower degree of nitrate in humans^16^^,^^22^^,^^38^^,^^39^. Although nitrite, nitrate and bicarbonate had not been measured in the urine, Pickerodt et al.^13^ concluded, solely on the basis of the plasma nitrite concentration course, which in fact differed between the groups, that acetazolamide did not alter renal function including CA-dependent reabsorption of nitrite and nitrate. As far as we are informed, plasma nitrite concentrations of the order of 35 µM and 48 µM as found at 1st h of hypoxia^13^ have not been reported until the present day. At such high concentrations the activity of many enzymes including erythrocytic catalase are likely to be inhibited to a considerable degree thus increasing oxidative stress and methemoglobinemia^40^.

In healthy subjects (mean weight, 60.7 kg), iNaNO_2_ (75 mg, 1.09 mmol) for 10 min resulted in a 20-fold increase of the mean blood nitrite concentration (from 0.2 µM to 4 µM) which decreased with an elimination half-life of 0.6 h^41^. The average FENO increased from 10 ppb at baseline to 40 ppb and fell to the baseline levels 10 min after NaNO_2_ inhalation^41^. No significant changes of systolic and diastolic pressure were observed in the human study, in which the iNaNO_2_ dose was 1.2 mg/kg body weight, i.e. much lower compared to the dose of 18.8 mg/kg body weight used in the study by Pickerodt et al.^13^. The results of the studies by Pickerodt et al.^13^ and Sirirat et al.^41^ indicate a very rapid elimination of iNaNO_2_ in accordance with an elimination half-life of about 0.5 h in humans^37^. The nitrite bioavailability of iNaNO_2_ in healthy subjects was reported to be 18%^41^. Unfortunately, neither nitrite nor nitrate excretion rates in the urine were reported in the studies by Pickerodt et al.^13^ and Sirirat et al.^41^. Such data would have allowed evaluating the expected drastically elevated excretion of nitrite due to inhibition of renal CA activity by acetazolamide in the animal study, as observed by us in humans at a pharmacological dose of acetazolamide^16^^,^^22^^,^^39^.

In 16 healthy young subjects (mean age, 23 years) who received intravenous infusion of NaNO_2_ (approximate dose, 0.48 mg/kg body weight) for 2 h after a 4-days pharmacological treatment period with placebo (only NaNO_2_, no drug) as control, allopurinol to inhibit xanthine oxidoreductase (XOR) activity, enalapril to inhibit angiotensin converting enzyme (ACE) activity, or acetazolamide (250 mg thrice a day) to inhibit CA activity, consistent results with respect to many parameters were obtained with all medications^6^. The authors concluded that the effects observed in their study, including the decrease of mean arterial pressure (−1.88 mmHg by placebo; −1.84 mmHg by allopurinol; −2.07 mmHg by enalapril; −2.32 mmHg by acetazolamide) suggest that XOR, ACE and CA are not essential for nitrite bioactivation to NO^6^. Yet, this conclusion is not convincing for the following reasons: the NaNO_2_ dose was low and the infusion period was relatively long when compared with the short elimination half-life of nitrite of 0.6 h; also the greatest effect of acetazolamide on nitrite excretion is quite rapidly exerted^16^^,^^22^^,^^39^.

A sophisticated animal study was performed by Wang et al.^14^. In CA II^+/+^, CA II ± and CA II^-/-^ mice (26 − 32 g body weight), infusion of NaNO_2_ (30, 50, 100, 500, 2500 nmol over 5 min corresponding to doses of up to 3.8 mg nitrite/kg body weight) lowered mean arterial blood pressure equally from about 70 mmHg down to 40 mmHg in CA II^+/+^, CA II ± and CA II^-/-^, suggesting a mechanism independent of CA II^14^. Unfortunately, the authors did not report on the possible formation of *S*-nitrosothiols, which could have been an indication of hCA II involvement. It is worth mentioning that increase in plasma *S*-nitrosothiol concentration was observed upon iNaNO_2_ for 10 min in doses of 0.06 to 2.2 mg NaNO_2_/kg body weight in healthy subjects^37^. Our group provided unequivocal evidence of the formation of ^15 ^N-labelled *S*-nitrosoglutathione (GS^15^NO) from ^15 ^N-labelled nitrite and GSH by means of a commercially available recombinant human erythrocytic CA II (heCA II) in Tris buffer at pH 7.4 in the absence of externally added bicarbonate^2^. In washed human platelets, the activity of sGC upon incubation with 100 µM nitrite, 20 mM bicarbonate and bovine erythrocytic CA II was comparable with that observed with 1 µM *S*-nitrosocysteine (CysSNO), one of the strongest endogenous inhibitors of platelet aggregation^29^. In the present study, comparable effects were obtained with 10 µM nitrite and 100 µM L-cysteine. This may suggest that 10 µM nitrite/100 µM L-cysteine is about 10 less active that SNP regarding NO formation.

Although CAs are investigated for several decades by many groups from various perspectives including biology and pharmacology, the CA family conceals many secrets and surprises^23^ that remain to be revealed. While there is solid evidence of the participation of renal CA II and CA IV in the reabsorption of endogenous and exogenous nitrite^16^^,^^39^, the mechanisms underlying the bioactivation by CA II of nitrite to species such as N_2_O_3_ with the potential of developing NO-related activity are still elusive and warrant further research. In vitro, haemoglobin species, XOR, ACE and CA have been shown to bioactivate nitrite under some specific conditions which included hypoxia and slightly acidic pH. The use of inhibitors of hCA II, XOR and ACE in studies on the bioactivation of nitrite is problematic for several reasons. The nitrous anhydrase activity of CA II and its importance in the bioactivation of inorganic nitrite to NO and *S*-nitrosothiols warrants further characterisation. Pharmacological acetazolamide exerts dilatory effects by not yet well-understood mechanisms, presumably in part independent of NO/cGMP^23^. The underlying mechanisms need elucidation before acetazolamide and other CA inhibitors can be used to test the involvement of hCA II in the bioactivation of nitrite. In this context the possibility should be considered that the use of hCA II inhibitors may result in loss of nitrite due to elevated renal excretion in the urine thus counteracting its bioactivation in other tissues. Measurement of urinary nitrite excretion in in vivo studies investigating the bioactivation of inorganic nitrite by CAs is mandatory. Lack of elevated urinary nitrite excretion and of other measures in the CA II^-/-^ mice^14^ could be due to alterations of other CA isozymes and proteins such as renin and aquaporin^42–44^.

### Are Zn- and copper-containing carbonic anhydrases (CuZnCA) responsible for contradictory results regarding nitrite bioactivation?

Cu^2+^ ions were found to bind to Zn^2+^-CA II isolated from human erythrocytes at a site other than the active site and to inhibit the exchange of water from the enzyme without affecting the equilibrium rate of hydration of CO_2_ by Tu et al. ^45^ almost four decades ago. This observation may suggest that classical CA inhibitors such as acetazolamide may inhibit the carbonic anhydrase activity of CA by tightly binding to CA II-bound Zn^2+^, but not to the second Cu^2+^-binding site. This may be an explanation for our previous^2^^,^^3^ and present (Figure 3) observations that neither acetazolamide nor dorzolamide inhibited the nitrous anhydrase activity of isolated bovine and human CA II.

The results of the present study observed with Cu-S^15^NALB and Cu-ALB suggest that L-cysteine is required for the bioactivation of the *S*-nitroso group of *S*-nitrosoalbumin (SNALB) either containing firmly bound Cu^2+^ or in the presence of non-*S*-nitrosylated but Cu^2+^ bound to albumin (Cu-ALB). It is likely that similar effects and mechanisms may also occur in ZnCu-CA II and CuCu-CA II. Yet this remains to be demonstrated by experiments analogous to those performed with Cu-S^15^NALB and Cu-ALB in the present study.

Zn-hCA II that additionally contains Cu^2+^ arising from contaminations or from Cu^2+^-containing proteins/enzymes such as ceruloplasmin could act as a nitrite reductase. The dependence of the nitrous anhydrase/nitrite reductase activity of CA II upon L-cysteine would be compatible with these particular activities. Reaction of L-cysteine with N_2_O_3_ in the bulk would form CysSNO (R8) of which the *S*-nitroso group is reduced to NO by Cu^1+^ ions (R11) formed from the reaction of Cu^2+^ with L-cysteine (R12). The so-called copper carbonic anhydrase has been in the past^46^^,^^47^ and is currently^48–51^ of particular interest not least because of its potential nitrite reductase activity. Reactions analogous to (R11) and (R12) could be formulated for zinc/copper human carbonic anhydrase II ([Zn^2+^/Cu^2+^]hCA II) (R13, R14). Based on a crystallographic study, it has been proposed that [Cu^2+^/Cu^2+^]hCA II is a nitrite reductase^50^^,^^51^, but no experimental evidence has been reported that nitrite is indeed reduced to NO by [Cu^2+^/Cu^2+^]hCA II or [Zn^2+^/Cu^2+^]hCA II) (R15)^52^. It is also unknown whether L-cysteine or ascorbic acid is required as reductants for Cu^2+^ bound to hCA II.

(R11) $$\text{Cys}-S-N=O+{\text{Cu}}^{1+}\to \text{Cys}-S^{-}+N^{•}=O+{\text{Cu}}^{2+}$$

(R12) $${\text{Cu}}^{2+}+\text{Cys}-S-H\to {\text{Cu}}^{1+}+\text{Cys}-S^{•}+H^{+}$$

(R13) $$\left(\left[{\text{Zn}}^{2+}/{\text{Cu}}^{1+}\right]\right)\text{hCA}\;\text{II}-{\text{Cys}}^{205}-S-N=O\to \left(\left[{\text{Zn}}^{2+}/{\text{Cu}}^{2+}\right]\right)\text{hCA}\;\text{II}-{\text{Cys}}^{205}-S^{-}+N^{•}=O$$

(R14) $$\left(\left[{\text{Zn}}^{2+}/{\text{Cu}}^{2+}\right]\right)\text{hCA}\;\text{II}-{\text{Cys}}^{205}-S-H+\text{Cys}-S-H\to \left(\left[{\text{Zn}}^{2+}/{\text{Cu}}^{1+}\right]\right)\text{hCA}\;\text{II}-{\text{Cys}}^{205}-S^{-}+\text{Cys}-S^{•}+H^{+}$$

(R15) $$\left(\left[{\text{Zn}}^{2+}/{\text{Cu}}^{1+}\right]\right)\text{hCA}\;\text{II}-{\text{Cys}}^{205}-S-H+O=N-O^{-}\to \left(\left[{\text{Zn}}^{2+}/{\text{Cu}}^{2+}\right]\right)\text{hCA}\;\text{II}-{\text{Cys}}^{205}-S-H+N^{•}=O$$

Human serum albumin (HSA) contains two specific Cu^2+^ binding sites, the N-terminal site (NTS) and the multimetal binding site (MBS). The NTS binds Cu^2+^ ions much more strongly than the MBS, and it is therefore considered to be the only HSA site ever occupied by Cu^2+^ ions in blood serum. In vitro, the incorporation of Cu^2+^ into HSA is fast. The affinity of Cu^2+^ to the NTS of HSA is 1 pM^53^. Under physiological conditions only about 1% of HSA molecules are estimated to carry a Cu^2+^ ion. About 25% of HSA molecules derived from human blood have their N-terminal dipeptide clipped and do not possess an NTS. The NTS is composed of the first three amino acid residues of the HSA sequence: Asp-Ala-His. In our study we did not determine the concentrations of Cu-S^15^NALB and Cu-ALB. We consider that they correspond to the measured protein concentrations. Externally added Cu^2+^ ions are likely to be bound on the NTS of our synthesised and purified Cu-S^15^NALB and Cu-ALB preparations, with no appreciable mutual hindrance and fully accessibility for L-cysteine. It is possible that Cu^2+^-containing CA may act as a nitrite reductase analogous to Cu^2+^-bound HSA, but this remains to be demonstrated in forthcoming experiments.

## Conclusions and perspectives

Several endogenous and exogenous sources contribute to inorganic nitrite. Do bovine and human carbonic anhydrase isoforms, notably CA II, possess nitrous anhydrase activity or nitrite reductase activity like bacterial nitrite reductases or possibly both? These questions are currently in the focus of scientific research due to the potential bioactivation of inorganic nitrite to nitric oxide (NO) and nitrous anhydride (N_2_O_3_). NO is a potent vasodilatator and inhibitor of platelet aggregation, which are considered to be beneficial in certain cardiovascular diseases. N_2_O_3_ can nitrosate numerous biomolecules thus potentially contributing to health via *S*-nitrosation and subsequent NO release, or to disease such as cancer via *N*-nitrosation. Existing results from *in vitro* and in vivo experiments in animals and humans including the effects of established and novel carbonic anhydrase inhibitors, including those for treatment of hypoxic tumours,^54^ are contradictory, often misinterpreted (discussed in References^52^^,^^55^), and difficult to reconcile. The lack of an inhibitory effect of acetazolamide on nitrous anhydrase/nitrite reductase activity of carbonic anhydrase is often interpreted as a CA-independent effect. The potentiation of the effects of CA on nitrite by L-cysteine seen in human washed platelets in our study is compatible with nitrous anhydrase activity of intra-platelet CA accompanied by subsequent NO release. This can be concluded because measuring of VASP phosphorylation is considered to reflect platelet-derived NO^56^. This effect may simulate “nitrite reductase” activity of CA. Yet, due to the ability of CA to bind Cu^2+^ on a site distinctly different from the Zn^2+^ site, further investigations using sophisticated analytical approaches, such as GC-MS in combination with stable-isotope labelled water and nitrite^57^, and proteomics, are required to reveal hidden biochemical properties of CA II and other CA-isoforms beyond their inherent carbonic anhydrase activity (R1). *S*-Glutathionylation of CA VII was found not to be associated with loss of catalytic activity and affinity for sulphonamide inhibitors^58^. Such investigations are required for Zn-hCA II regarding the involvement of Cys^205^ in the nitrous anhydrase activity, and for Cu-hCA II regarding the involvement of Cys^205^ in its proposed nitrite reductase activity.
